# Surgeon practices regarding infection prevention for growth friendly spinal procedures

**DOI:** 10.1007/s11832-014-0584-1

**Published:** 2014-04-18

**Authors:** Michael P. Glotzbecker, Sumeet Garg, Behrooz A. Akbarnia, Michael Vitale, Tricia St Hillaire, Ajeya Joshi

**Affiliations:** 1 Department of Orthopaedic Surgery, Boston Children’s Hospital, Harvard Medical School, 300 Longwood Avenue, Hunnewell 2, Boston, MA 02115 USA; 2 Department of Orthopaedic Surgery, University of Colorado, Denver, CO USA; 3 San Diego Center for Spinal Disorders, San Diego, CA USA; 4 Department of Orthopaedic Surgery, Columbia University, New York, NY USA; 5 Shriners Hospital for Children, Philadelphia, PA USA; 6 Children’s Hospital of San Antonio, San Antonio, TX USA

**Keywords:** Spinal infection, Growth friendly surgery, Early onset scoliosis, Infection prevention, Infection reduction

## Abstract

**Purpose:**

The rate of infection in patients having growth sparing surgery for early onset scoliosis has been reported up to 25 % during the course of treatment. A recent study demonstrated significant variability in the approach to infection prevention in adolescent and neuromuscular scoliosis. The purpose of this study is to conduct a similar survey in order to understand approaches used by experienced pediatric spinal surgeons with regard to infection prevention in growth friendly spinal procedures.

**Materials and methods:**

After preliminary internal testing of a survey by the authors, a final 21-question survey was created and approved by the authors and electronically distributed to all members of the Chest Wall Spinal Deformity Study Group and the Growing Spine Study Group (*n* = 57). A total of 40 responses were obtained (70 %).

**Results:**

Significant variability in practice was demonstrated across the majority of the questions answered. Several of the questions demonstrated relative equipoise between practices, including preoperative MRSA screening, preoperative chlorhexidine baths, postoperative antibiotic duration after insertion, use of topical antibiotics, use of drains, use of IV gram negative coverage or vancomycin, and skin preparation.

**Conclusion:**

Other studies have demonstrated that variability in practice may have a negative impact on clinical outcomes, so one could postulate that steps that can reduce variability in the current population may help improve outcomes in this population. Areas of clinical equipoise can be used to help design and direct multicenter studies with an ultimate goal of reducing infections in this population.

**Level of evidence:**

Level V.

**Electronic supplementary material:**

The online version of this article (doi:10.1007/s11832-014-0584-1) contains supplementary material, which is available to authorized users.

## Introduction

The consequences of a postoperative spinal infection include significant direct and indirect costs [1]. The rate of infection in patients after growth sparing surgery for early onset scoliosis (EOS) has been reported in up to 25 % of patients during the course of treatment (unpublished data) [2–4]. Surgical site infections are associated with prolonged hospital stays and IV antibiotic regimens, and frequently require multiple reoperations for debridements and implant removal [1, 5, 6].

As demonstrated by a recent systematic review of the literature, there is a lack of well-designed trials and evidence in the pediatric literature to help guide infection prevention strategies after pediatric deformity surgery in the adolescent and neuromuscular populations [7]. With a lack of good evidence, attempts to adopt best practice are unfortunately often dictated by personal experience. This leads to significant variability in practice patterns in the approach to infection prevention in deformity surgery [8].

Understanding current surgical practices, as well as the current literature, are initial steps required to reduce variability of practice through the development and adoption of best evidence guidelines [9]. Such guidelines can improve clinical outcomes and reduce healthcare costs [10–12]. While a recent survey demonstrated significant variability in adolescent idiopathic and neuromuscular surgery [8], the growing spine population is unique with regard to underlying diagnosis, risk factors, and surgical techniques. The purpose of this study is to understand approaches used by experienced pediatric spinal surgeons with regard to infection prevention after growth friendly spinal procedures.

## Materials and methods

After creation and preliminary internal testing of a survey by the authors, a final 21-question survey was approved by the authors and electronically distributed to all 57 members of the Chest Wall and Spinal Deformity Study Group and the Growing Spine Study Group (*n* = 57). Questions focused on current practices involving infection prevention when using growth friendly implants, and included practice patterns during initial insertion as well as after lengthening procedures. The questions were developed through discussion by the authors, and were derived from techniques currently commonly used in early onset and adolescent populations [7–9]. The basis of the current survey was a similar survey considering infection prevention practices in high risk (neuromuscular) populations [8]. While many questions were similar in the two surveys, different questions were required for this survey as the population and the surgeries used in this population are associated with unique issues. Each question was in multiple choice format and was followed by 2–8 possible responses. When appropriate, an open ended response “other (please specify)” was included to accommodate additional comments or choices that were not listed as options (Supplementary Appendix 1). It took approximately 5 min to complete, and 40 responses were obtained (70 %).

### Source of funding

No external funding source was required for the current study. No funding was received for this work from any of the following organizations: National Institutes of Health (NIH); Welcome Trust; Howard Hughes Medical Institute (HHMI).

### Statistical methods

No statistical analysis was needed for the current study.

## Results (Tables 1, 2)

### Insertion Procedures

Of the respondents, 46.1 % of surgeons use chlorhexidine baths at home preoperatively for insertions, 42.5 % obtain preoperative laboratories (such as albumin, prealbumin, TWBC, TLC, serum transferrin) to stratify for infection, and 30.8 % obtain MRSA swabs to guide preoperative antibiotic choice.

**Table 1:** Summary of survey results to questions asked including most common and other methods used

Intervention	Most commonly used (% used)	Other methods used (% used)
Preoperative prophylactic antibiotics		
Insertion	IV cephalosporin (74.4)	IV vancomycin vs. cephalosporin based on MRSA screen (17.9)
		Vancomycin (5.1)
		Clindamycin (5.1)
		Gram-negative (12.8)
		Other (10.2)
Lengthening	IV cephalosporin (74.4)	IV vancomycin vs. cephalosporin based on MRSA screen (15.4)
		IV vancomycin (2.6)
		Clindamycin (5.1)
		Gram-negative (2.6)
Skin preparation		
Insertion	Chloraprep^®^ (61.5)	Alcohol (38.5)
		Betadine (23.1)
		Duraprep^®^ (23.1)
		Other (15.4)
Lengthening	Chloraprep^®^ (60.5)	Alcohol (36.8)
		Betadine (23.7)
		Duraprep^®^ (23.7)
		Other (15.8)
Wound irrigation		
Insertion	Saline (74.4)	Dilute betadine (17.9)
		Bacitracin (15.4)
		Other (7.7)
Lengthening	Saline (84.2)	Dilute betadine (13.2)
		Bacitracin (7.9)
		Other (7.9)
Topical antibiotics		
Insertion	Vancomycin (41)	None (48)
Lengthening	n/a	
Dressing		
Insertion	Impervious seal dressing (59)	Nonadherent dressing (28.2)
		Standard seal tape (20.5)
		Padding (25.6)
		Other (25.6)
Lengthening	n/a	
Duration postoperative antibiotics		
Insertion	IV antibiotics <24 h (64.1)	IV antibiotic >24 h (33.3)
		Only a single preoperative dose (2.6)
Lengthening	IV antibiotics <24 h (47.4)	None (47)
		IV antibiotic >24 h (2.6)
		Oral antibiotics >24 h (2.6)

Certain questions allowed for choosing more than one selection so the total percentage for each question does not equal 100 %

*Chloraprep*^*®*^ chlorhexidine gluconate, *duraprep*^*®*^ iodine povacrylex and isopropyl alcohol, *betadine* povidone iodine

**Table 2:** Summary of survey results to questions asked including most common and other methods used

Intervention	Percentage used
Chlorhexidine baths	
Insertion	46.1
Lengthening	41
Preoperative nutrition labs	
Insertion	42.5
Lengthening	5
Preoperative MRSA swab	
Insertion	30.8
Lengthening	n/a
Drains	
Insertion	41.1
Lengthening	2.6

When choosing preoperative prophylactic antibiotics, 74.4 % use intravenous (IV) cephalosporin, 17.9 % use IV vancomycin or cefazolin based on the results of the preoperative MRSA nasal swab culture, 5.1 % use IV vancomycin, 5.1 % use clindamycin, and 12.8 % use gram negative coverage (gentamycin). Other (10.2 %) responses included using gram negative coverage for incontinent or high-risk patients. Of surgeons using antibiotics, 64.1 % use them for 24 h or less, while 33.3 % continue prophylactic antibiotics for >24 h, and 2.6 % use only a single preoperative dose.

**Fig. 1:**
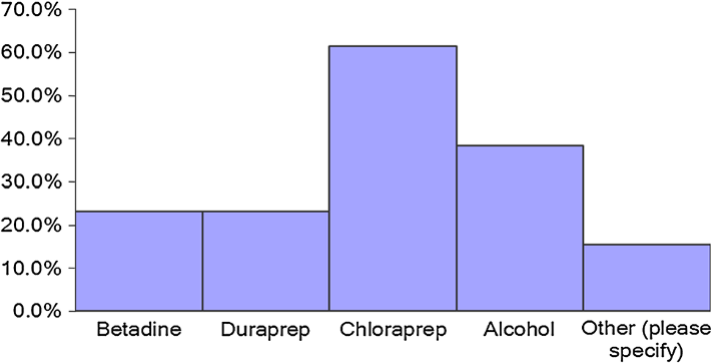
Graphical depiction of variability in skin preparation prior to surgery amongst surgeons surveyed

Skin preparation used for insertion procedures (surgeon allowed to choose more than one) included betadine (povidone iodine) in 23.1 % of patients, duraprep^®^ (iodine povacrylex and isopropyl alcohol) in 23.1 %, chloraprep^®^ (chlorhexidine gluconate) in 61.5 %, alcohol in 38.5 %, and other modalities in 15.4 %. Other responses included using scrub or soap prior to other prep solutions (Fig. 1). Regarding incisions, 57.9 % of surgeons make a separate fascial incision from the skin incision for both VEPTR^®^ and growing rods, 28.9 % do it for VEPTR^®^ procedures only, and 2.6 % do it for growing rods procedures only; however, 10.5 % do not make a separate fascial incision from the skin incision. Traffic is limited in the operating room during the procedure by 63.2 % of surgeons surveyed.

For irrigating the wounds prior to closure, 66.7 % of surgeons use a bulb syringe, 28.2 % use pulse lavage, and 5.1 % use cysto tubing. For an insertion procedure, 74.4 % use saline, 15.4 % use bacitracin and saline, 17.9 % use dilute betadine, and 7.7 % use other fluids such as kefzol or triple antibiotics for their intraoperative irrigant. For an insertion procedure, 41 % use intraoperative topical vancomycin in the wound/bone graft, while 48 % do not use intraoperative antibiotics. Drains are used by 41.1 % of surgeons for insertion procedures. For a postoperative dressing, 28.2 % use moist nonadherent dressing (adaptic, xeroform, etc.), 20.5 % use standard seal tape (silk, paper, metapore, etc.), 59 % use impervious seal dressing, 25.6 % use padding, and 25.6 % use other dressings such as silver impregnated dressings, dermabond, or mepilex.

### Lengthening procedure

41 % of surgeons use chlorhexidine baths at home prior to a lengthening procedure. Five percent of surgeons obtain preoperative labs prior to a lengthening procedure. 74.4 % use IV cephalosporin, 2.6 % use IV vancomycin, 5.1 % use clindamycin, and 2.6 % use gram negative coverage (gentamycin) as standard preoperative antibiotic prior to a lengthening procedure. 15.4 % use IV vancomycin or cefazolin based on a preoperative MRSA culture. Other responses included using gram negative coverage for incontinent or high risk patients. 47 % do not give postoperative antibiotics after a lengthening procedure, 47.4 % continue them for 24 h or less, 2.6 % continue IV antibiotics for >24 h, and 2.6 % use oral antibiotics for >24 h. Skin preparation used for lengthening procedure include betadine (23.7 %), duraprep^®^ (23.7 %), chloraprep^®^ (60.5 %), alcohol (36.8 %), and other (15.8 %). Other responses included using scrub or soap prior to other prep solutions. For a lengthening procedure, 84.2 % of surgeons use saline as an intraoperative irrigant while 7.9 % use bacitracin, 13.2 % use dilute betadine, and 7.9 % responded “other.” Only 2.6 % of surveyed surgeons use drains for lengthenings.

### Postoperative infection

**Fig. 2:**
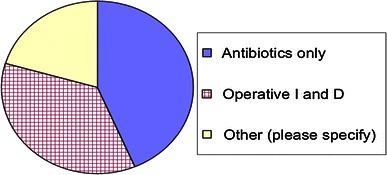
Variable approach of surgeons toward a superficial infection

When asked how they would treat a superficial infection, 43.6 % of surgeons treat superficial infection with antibiotics only while 35.9 % treat with operative incision and drainage (Fig. 2).

## Discussion

The current study demonstrates that there is significant variability in practice regarding infection prevention measures in the growing spine population. This survey was conducted amongst active members of two research groups that work together frequently, are experienced with growth friendly procedures, and are heavily invested in improving the care of these children. Despite this group’s relative familiarity of the current literature and treatment trends, there was a significant divergence of opinions on multiple questions. Given the experience of the surgeons surveyed, one might postulate that using the physicians surveyed in these groups may actually underestimate the true variability in practice.

Patients with EOS encompass a diverse population and include multiple diagnoses (idiopathic, congenital, neuromuscular, syndromic). Therefore, it is probably unreasonable to suggest that a uniform blanket approach can be applied to this population. Given the cost of an infection to both the family and healthcare, understanding processes that can reduce the risk of an infection is important. A recent study (unpublished data) and several other published studies have demonstrated a risk of infection in this population of up to 25 % [2–4]. Given the high incidence of infection associated with these growth friendly procedures, any measure that reduces this risk will have substantial clinical importance.

Variability in medical practice is common, and most studies suggest that efforts which reduce variability and encourage adoption of best practice can have a positive impact on both clinical outcome and healthcare cost [10–12]. The orthopaedic literature, and specifically literature related to preventing spinal infection, is largely comprised of retrospective case series and expert opinion, rather than true randomized clinical trials (RCTs) [13]. There are considerable barriers to completing well-designed clinical trials in orthopaedics; however, such studies are required to improve our clinical processes [13]. With a lack of available good evidence, surgeons are faced with a difficult challenge to adopt best practices based on personal experience.

While there is literature to suggest that measures such as preoperative MRSA screening [14–16], chlorhexidine skin preparation [17], use of dilute povidone iodine solution prior to closure [18–21], and the use of gentamycin or vancomycin in the bone graft after spinal fusion [22–27] may reduce infection in some patients, it is not clear that these studies can be generalized outside of the populations studied. While the presence of this literature encourages some to adapt these practices to the growing spine population, it is not obvious that these interventions will have similar outcomes in a much different patient population. It is not surprising, given the diversity of the growing spinal deformity population, and the lack of available evidence that there is such variability in practice. While unreasonable to expect that all variability can be eliminated, we can postulate from other studies that any measures which reduce variability and lead to standardized processes may have a positive impact on this population.

While many factors such as skin preparation and wound irrigation are similar between the two lengthening and insertion groups, it should be noted that there are some key differences with regard to infection prevention strategies for a lengthening procedure. This is likely due to several factors such as shorter operative time and smaller incisions, and that it is often a soft tissue procedure and does not involve direct contact with the bone. This likely explains the less frequent use of drains and intraoperative antibiotics, as well as different strategies with regard to postoperative prophylaxis.

Often the best we can do is to collect the available information and to synthesize it in a systemic manner. This process requires identifying current practice and comparing it to the available literature. Once this is done, the information can be used to try to create best practice guidelines, and to serve as a platform for designing prospective clinical studies. This process has recently been proven effective for high-risk spinal patients [7–9], and therefore this study represents the first step to adopting similar methodology to the growing spine population. However, the individual surgeon may use these results and choose methods in which there is a majority opinion. While the majority opinion may not be data driven, it offers opportunities to reduce variability within one’s individual and institutional process.

In addition to initiating a discussion that can be used to develop best practice guidelines, this study can also be used to design studies moving forward. For example, there were a number of study questions identified in the current study where there is sufficient equipoise to design clinical trials. Questions with a fairly diverse/even distribution of answers in this study included the use of drains in insertion procedures, using perioperative IV vancomycin or gram negative coverage, postoperative antibiotic duration after insertions and lengthenings, preoperative skin preparation, antibiotic use in bone graft, preoperative MRSA screening, and preoperative chlorhexidine baths.

There are several limitations to the current study. While the response rate was fairly high, the physicians surveyed represent a somewhat homogeneous, experienced population in that they have a specific research interest in this population. Further, while we may postulate that variability is bad for clinical care, the effect of variability in infection prevention after surgery for EOS is unproven. Finally, this study is essentially expert opinion, and only represents a summary of surgeon opinions. The purpose here is to demonstrate the current variability of practice, and is not meant to suggest which treatments may or may not reduce infection rates, as that is outside the scope of the current paper.

## Conclusion

There is significant variability of current practices of surgeons who perform surgery for EOS. Variability in medical practice is probably not ideal, and measures which can reduce this variability may have a positive impact on both patient outcomes and health care efficiency. Identifying majority opinion practices within this study can be a starting point to reducing individual and institutional variability. Further, this study is the first step in identifying best practices as well as potential topics for multicenter collaborative research.

## Electronic supplementary material

Below is the link to the electronic supplementary material.

Appendix 1: Surgeon Practices Regarding Surgical Site Infection (SSI) Prevention Form

Supplementary material 1 (PDF 121 kb)
