# Supplementary material for: Surgeon practices regarding infection prevention for growth friendly spinal procedures
Source: J Child Orthop. 2014 Apr 18;8(3):245–50. doi: 10.1007/s11832-014-0584-1 (PMC4142892; doi:10.1007/s11832-014-0584-1)
Supplement: Supplementary file 1 — Supplementary material 1 (PDF 121 kb) [file 11832_2014_584_MOESM1_ESM.pdf]

Surgeon Practices Regarding Surgical Site Infection (SSI) Prevention Form

Infection is poorly studied in patients with early onset scoliosis (EOS) who are being treated with growth friendly strategies. Unfortunately, the infection risk in these patients is as high as 25%. There is considerable variability of current practices of surgeons who perform deformity surgery with regard to infection prevention. The purpose of this survey is to understand the approaches currently used amongst pediatric spinal surgeons who treat patients with EOS.

Regarding your approach to a pediatric patient undergoing spinal surgery for EOS (ie Growing Rods, VEPTR, or other techniques requiring repetitive lengthening):

1. Do you ask patients to do anything at home preoperatively?

- [ ] No
- [ ] Yes, chlorhexidine all patients
- [ ] Yes, chlorhexidine INSERTION procedure ONLY
- [ ] Yes, Other ______________________ (please specify)

2. Do you obtain any preoperative labs to stratify for infection (ie: albumin, prealbumin, TWBC, TLC, serum Transferrin)?

- [ ] Yes, all patients
- [ ] Yes, INSERTION procedure ONLY
- [ ] No
- [ ] Other ______________________ (please specify)

3. Do you obtain preoperative surveillance cultures (ie MRSA swabs) to guide preoperative antibiotic choice?

- [ ] Yes
- [ ] No

4. What is your standard preoperative antibiotic choice for an INSERTION procedure?
(YOU MAY SELECT MORE THAN ONE)

- [ ] IV Cephalosporin
- [ ] IV Vancomycin
- [ ] IV Vancomycin or Cefazolin
- [ ] (based on MRSA culture and/or age)
- [ ] IV Clindamycin
- [ ] IV Gram negative coverage *(ie gentamycin)*
- [ ] Other ______________________ (please specify)

5. What is your standard preoperative antibiotic choice for a LENGTHENING procedure?
(YOU MAY SELECT MORE THAN ONE)

- [ ] IV Cephalosporin
- [ ] IV Vancomycin
- [ ] IV Vancomycin or Cefazolin
- [ ] (based on MRSA culture and/or age)
- [ ] IV Clindamycin
- [ ] IV Gram negative coverage *(ie gentamycin)*
- [ ] No antibiotic for simple lengthening
- [ ] Other ______________________ (please specify)

6. How long do you continue antibiotics use after index growth friendly procedure?

- [ ] Preoperative dose only
- [ ] 24 hours or less
- [ ] Greater than 24 hours

7. How long do you continue IV antibiotics after simple lengthening procedure?

- [ ] No postoperative antibiotics given
- [ ] 24 hours or less
- [ ] Greater than 24 hours
- [ ] Oral antibiotics given for 24 hours or less
- [ ] Oral antibiotics given for greater than 24 hours

8. What is your standard skin preparation for an INSERTION procedure? (YOU MAY SELECT MORE THAN ONE)

- [ ] Betadine
- [ ] Chloraprep
- [ ] Alcohol
- [ ] Duraprep
- [ ] Other ______________________ (please specify)

9. What is your standard skin preparation for a LENGTHENING procedure? (YOU MAY SELECT MORE THAN ONE)

- [ ] Betadine
- [ ] Chloraprep
- [ ] Alcohol
- [ ] Duraprep
- [ ] Other ______________________ (please specify)

10. Do you limit traffic in your operating room?

- [ ] Yes
- [ ] No

11. Do you make a separate fascial/muscular incision that is not directly beneath the skin incision?

- [ ] Yes, VEPTR ONLY
- [ ] Yes, Growing Rods ONLY
- [ ] Yes, VEPTR & GR
- [ ] No

12. What is your standard intraoperative irrigation delivery system?

- [ ] Bulb syringe Saline
- [ ] Pulse lavage Saline
- [ ] Cysto tubing Saline

13. What is your standard postoperative irrigation solution for an INSERTION procedure? (YOU MAY SELECT MORE THAN ONE)

- ☐ Saline
- ☐ Soap
- ☐ Bacitracin
- ☐ Dilute Betadine
- ☐ Other ____________________ (please specify)

14. What is your standard postoperative irrigation solution for a LENGTHENING procedure? (YOU MAY SELECT MORE THAN ONE)

- ☐ Saline
- ☐ Soap
- ☐ Bacitracin
- ☐ Dilute Betadine
- ☐ Other ____________________ (please specify)

15. Do you use Antibiotics in your bone graft or in the wound for an INSERTION procedure?

- ☐ Yes-Gentamycin
- ☐ Yes-Vancomycin
- ☐ No
- ☐ Other ____________________ (please specify)

16. Do you use drains?

- ☐ Yes, all patients
- ☐ Yes, INSERTION only
- ☐ No
- ☐ Variable (please specify)

17. Rank each in level of importance from 1 (not important) to 5 (very important) the factors you feel most important in minimizing the risk of a postoperative infection

	1 (not important)	2	3	4	5 (very important)
preop co-morbidities (including nutritional status)					
antibiotic choice					
antibiotic timing					
skin prep					
blood loss					
operative time					
type of implants used					
use of drains					
OR traffic					
antibiotic in bone graft					
Betadine irrigation					
UTI (Myelo) treatment preop					
quality of skin covering incision					
suture choice					

18. On your postoperative dressing, please indicate which of the following is used (YOU MAY SELECT MORE THAN ONE)

- ☐ Moist nonadherent dressing (adaptic, xeroform, etc)
- ☐ Standard seal tape (silk, paper, metapore, etc)
- ☐ Impervious seal dressing (tegaderm, ioban, mepilex, etc)
- ☐ Padding (foam, etc)
- ☐ Other ____________________ (please specify)

19. How do you treat a superficial infection (ie to prevent a deep infection?)

- ☐ Antibiotics only
- ☐ Operative I and D
- ☐ Other ____________________ (please specify)

20. Do you have any other strategies that you utilize to reduce infection in this population?

21. Comments
